# Exercise: a molecular tool to boost muscle growth and mitochondrial performance in heart failure?

**DOI:** 10.1002/ejhf.2407

**Published:** 2022-01-09

**Authors:** Kirsten T. Nijholt, Pablo I. Sánchez‐Aguilera, Suzanne N. Voorrips, Rudolf A. de Boer, B. Daan Westenbrink

**Affiliations:** ^1^ Department of Cardiology University Medical Centre Groningen, University of Groningen Groningen The Netherlands

**Keywords:** Heart failure, Exercise intolerance, Exercise training, Cardiac and skeletal muscle, Mitochondrial adaptation, Physiological muscle hypertrophy

## Abstract

Impaired exercise capacity is the key symptom of heart failure (HF) and is associated with reduced quality of life and higher mortality rates. Unfortunately, current therapies, although generally lifesaving, have only small or marginal effects on exercise capacity. Specific strategies to alleviate exercise intolerance may improve quality of life, while possibly improving prognosis as well. There is overwhelming evidence that physical exercise improves performance in cardiac and skeletal muscles in health and disease. Unravelling the mechanistic underpinnings of exercise‐induced improvements in muscle function could provide targets that will allow us to boost exercise performance in HF. With the current review we discuss: (i) recently discovered signalling pathways that govern physiological muscle growth as well as mitochondrial quality control mechanisms that underlie metabolic adaptations to exercise; (ii) the mechanistic underpinnings of exercise intolerance in HF and the benefits of exercise in HF patients on molecular, functional and prognostic levels; and (iii) potential molecular therapeutics to improve exercise performance in HF. We propose that novel molecular therapies to boost adaptive muscle growth and mitochondrial quality control in HF should always be combined with some form of exercise training.

## Introduction: the unmet need to alleviate exercise intolerance in heart failure patients

Exercise intolerance is the main clinical symptom of heart failure (HF) and a key factor contributing to the reductions in quality of life of those affected.^1^, ^2^, ^3^, ^4^, ^5^, ^6^ Exercise intolerance – defined as an inability to perform physical activity – is accompanied by symptoms such as dyspnoea on exertion and fatigue, and affects HF patients with preserved and reduced ejection fraction (HFpEF and HFrEF, respectively) to the same extent.^4^ While exercise intolerance in HF is primarily caused by diminutions in circulatory performance, the mechanisms are far more complex and also involve functional and structural abnormalities in cardiac and skeletal muscle as well as respiratory dysfunction and deconditioning.^4^

Contemporary HF therapies are designed to reduce hospitalizations and mortality. While many HF drugs that reduce mortality also influence exercise capacity, the magnitude of improvement is modest and variable.^4^, ^7^, ^8^, ^9^, ^10^, ^11^, ^12^, ^13^, ^14^, ^15^ Accordingly, exercise tolerance and activity levels of HF patients remain low.^5^, ^16^, ^17^, ^18^

On the other hand, evidence is emerging to suggest that exercise training can decrease morbidity and mortality in HF patients.^2^, ^5^, ^16^, ^19^, ^20^ This was recently confirmed in the REHAB‐HF trial in which an exercise intervention improved physical function.^20^ Exercise induces specific structural and molecular changes in HF patients that cause sustained improvements in exercise performance. Mechanistic insights into these pathways may uncover nodal points for therapeutic interventions to improve exercise performance in HF. With the current review, we provide an overview of exercise‐induced changes that may benefit HF patients, focusing on physiological signalling pathways and mitochondrial adaptations in cardiac and skeletal muscle.

## Physiological adaptation in cardiac and skeletal muscle in response to exercise

### Signalling pathways that govern physiological cardiac growth

**Figure 1:**
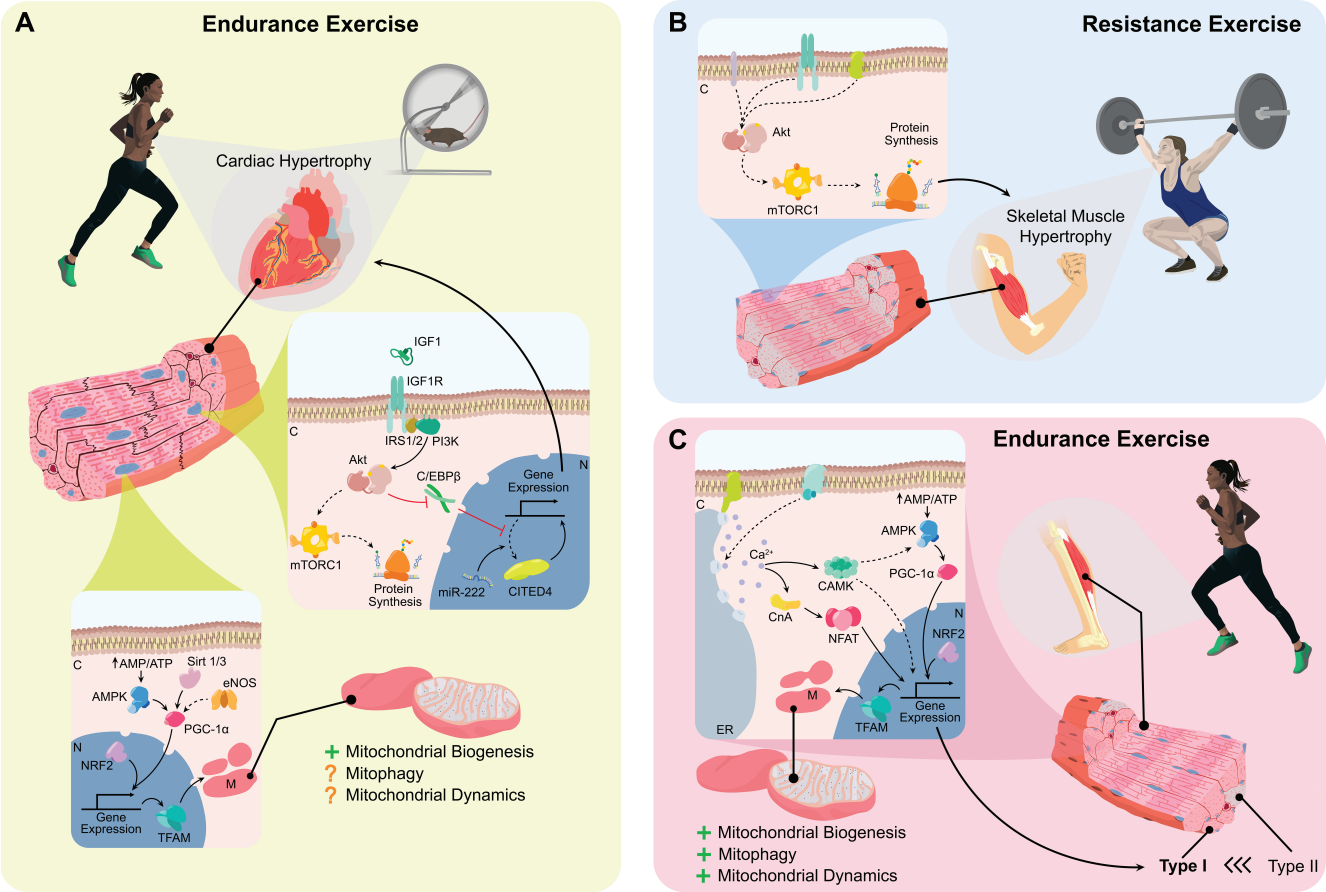
Physiological adaptation in cardiac and skeletal muscle. (*A*) The adaptive effects of endurance exercise on cardiac muscle are governed by special signal transduction pathways and mitochondrial quality control. Upper panel: exercise stimulates binding of insulin‐like growth factor 1 (IGF1) to a specific transmembrane tyrosine kinase membrane receptor (IGF1R), causing a conformational change that recruits and phosphorylates insulin receptor substrates 1 and 2 (IRS1/2). In turn, the activation of IRS1/2 phosphorylates phosphoinositide 3 kinase (PI3K) and further downstream activation of protein kinase B (Akt). The diverse effects of Akt activation include for example activation of endothelial nitric oxide synthase (eNOS), activation and/or inhibition of sirtuins, inhibition of glycogen synthase kinase 3β as well as inhibition of forkhead box protein O3 (FOXO3). Most importantly, however, activation of Akt subsequently (i) promotes protein synthesis through activation of mammalian target of rapamycin complex 1 (mTORC1), its downstream activation of ribosomal protein S6 kinase 1 (S6K1) and inhibition of eIF4E‐binding protein 1 (4EBP1) and (ii) by inhibiting the transcriptional repressor CCAAT/enhancer binding protein‐β (C/EBPβ) to activate a specific physiological growth programme downstream of the transcription factor CBP/p300‐interacting trans activator 4 (CITED4). Lower panel: exercise also enhances mitochondrial performance through enhanced mitochondrial biogenesis, and potentially also through increased mitochondrial clearance (mitophagy) and mitochondrial morphological changes (mitochondrial dynamics). Exercise stimulates mitochondrial biogenesis through activation of AMP‐activated protein kinase (AMPK) and upregulation of sirtuin 1/3 (SIRT1/3) and eNOS. These factors in turn promote the activity of the transcription factor peroxisome‐proliferator‐activated‐receptor gamma coactivator 1‐alpha (PGC‐1α) and its downstream factors nuclear respiratory factor 2 (NRF2) and mitochondrial transcription factor A (tFAM). NRF2 and tFAM are both essential for the generation of new mitochondrial proteins. (*B*) Resistance exercise induces skeletal muscle hypertrophy mediated through Akt, which activates mTORC1 leading to the synthesis of new proteins and muscle growth. (*C*) In skeletal muscle, endurance exercise causes an increase in mitochondrial biogenesis, mitophagy and mitochondrial dynamics. Mitochondrial biogenesis is regulated by activation of AMPK, PGC‐1α and downstream NRF2 as well as tFAM. An additional effect of exercise in skeletal muscle fibres includes a shift toward a more oxidative composition through calcineurin (CnA) mediated activation of nuclear factor of activated T‐cells (NFAT) and Ca^2+^/calmodulin‐dependent protein kinase (CaMK), or through modulation of AMPK and PGC‐1α. Full lines indicate direct effects, dashed lines indicate indirect effects. AMP, adenine nucleotide monophosphate; ATP, adenine nucleotide triphosphate; C, cytoplasm; Ca^2+^, calcium; ER, endoplasmic reticulum; M, mitochondria; miR‐222, microRNA; N, nucleus.

In 1801, Corvisart was the first to hypothesize that an increase in cardiac mass was the result of increases in cardiac workload.^21^ This hypothesis was confirmed by Kuelbs et al. who demonstrated that exercise in dogs resulted in an increase in cardiac mass, which was absent in sedentary controls.^22^ Many years later, a variety of experimental designs using multiple species – ranging from voluntary to forced exercise – have uncovered a panel of growth factors, intracellular signalling pathways and transcriptional responses that distinguish pathological from physiological cardiac growth.^23^, ^24^, ^25^, ^26^, ^27^, ^28^, ^29^ A critical event in the development of physiological hypertrophy is the release of the specific physiological peptide growth factors, including but not restricted to insulin‐like growth factor 1 (IGF1).^23^, ^29^ Binding of IGF1 to its surface receptor on cardiomyocytes activates the IGF1‐phosphoinositide 3 kinase‐protein kinase B (IGF1‐PI3K‐Akt) pathway that governs a plethora of adaptive changes (summarized in *Figure* 1*A*).^23^, ^29^, ^30^

It has been well established that the activation of the IGF1‐PI3K‐Akt pathway is required for physiological cardiac hypertrophy.^31^, ^32^, ^33^, ^34^, ^35^, ^36^, ^37^ For instance, cardiomyocyte‐specific overexpression of the IGF1 receptor (IGF1R) results in spontaneous physiological hypertrophy as well as a more profound increase in cardiac mass in response to swimming.^37^ Conversely, cardiac hypertrophy is attenuated after repetitive swimming exercises in cardiac‐specific IGF1R‐knock‐out mice.^36^ Pharmacological inhibition of PI3K‐Akt attenuates cardiac hypertrophy,^31^ whereas cardiac hypertrophy in response to swimming is blocked in Akt‐knock‐out mice.^35^ Further evidence for the critical importance of Akt has been established with a model using the Akt‐specific PH domain leucine‐rich repeat protein phosphatase (PHLPP1). In PHLPP1‐knock‐out mice, Akt phosphorylation is increased and physiological cardiac hypertrophy in response to swimming is augmented.^32^

The downstream effects of Akt that govern physiological cardiac growth are diverse,^19^, ^23^, ^29^, ^38^, ^39^ but two stand out as most clearly associated with exercise‐induced cardiac growth.^23^, ^39^ First, the mammalian target of rapamycin complex 1 (mTORC1) is phosphorylated by Akt, which subsequently leads to downstream activation of ribosomal protein S6 kinase 1 and inhibition of eIF4E‐binding protein 1 leading to protein synthesis and in turn cardiac hypertrophy.^23^, ^29^, ^39^ Second, the IGF‐1‐PI3K‐Akt signalling cascade was more recently also shown to regulate CCAAT/enhancer binding protein‐β (C/EBPβ), and CBP/p300‐interacting trans activator 4 (CITED4).^19^, ^23^, ^29^, ^39^ Akt phosphorylation downregulates cardiac C/EBPβ, which de‐represses the transcription factors serum response factor (SRF) and CITED4 and subsequently enables transcription of genes encoding for proteins that are critical for physiological cardiac hypertrophy.^19^, ^40^, ^41^, ^42^, ^43^, ^44^ Interestingly, in addition to hypertrophic growth pathways, CITED4 also appears to regulate ultrastructural changes and cardiomyocyte elongation.^19^, ^40^, ^42^, ^43^, ^45^ The role of CITED4 has been studied in a CITED4‐knock‐out mouse model, which was subjected to swimming and transaortic constriction.^44^ Physiological hypertrophy was attenuated, while pathological hypertrophy was exacerbated in CITED4‐knock‐out mice, indicating that CITED4 enables physiological growth while also preventing maladaptive responses. Interestingly, CITED4‐mediated protection is at least partially regulated by mTORC1.^44^ Other potential factors include microRNA‐222 (miR‐222) and miR30d.^19^, ^23^, ^40^, ^44^, ^46^, ^47^

### Exercise‐induced hypertrophy in skeletal muscle

While resistance exercise training induces robust hypertrophy in skeletal muscle – through mTORC1 – the hypertrophic response to endurance exercise is negligible. Instead, skeletal muscle increases the number of nuclei and satellite cells, adapts capillary and mitochondrial networks, and switches its muscle fibre type composition^48^, ^49^, ^50^, ^51^ (*Figure* 1*B*). Fast twitch (type II), glycolytic muscle fibres are favourable in anaerobic and resistance exercise, whereas slow twitch (type I), oxidative muscle fibres are preferable in aerobic endurance exercise as they contain more mitochondria.^48^ Skeletal muscle adapts to endurance exercise by switching towards more oxidative isoforms, primarily controlled by calcineurin signalling.^48^, ^49^, ^50^, ^51^, ^52^ Reprogramming towards oxidative isoforms is also associated with adaptive changes in mitochondrial quality control and function^48^, ^53^ (*Figure* 1*C*).

### Mitochondrial adaptations to exercise in cardiac muscle

Because exercise increases the demand for energy, mitochondrial numbers must increase as well^54^, ^55^, ^56^, ^57^, ^58^ (*Figure* 1*A*). *Mitochondrial biogenesis* refers to the generation of new mitochondria from existing organelles through the process of self‐replication,^54^, ^56^ and represents the principal mechanism responsible for exercise‐induced increases in mitochondrial content.^59^ Mitochondrial proteins are encoded by both mitochondrial and nuclear DNA, which requires accurate transcriptional regulation and protein import.^54^, ^56^, ^57^, ^60^ Several signalling pathways activate biogenesis including sirtuin 1 and 3 (SIRT1/3), AMP‐activated protein kinase (AMPK) and endothelial nitric oxide synthase (eNOS).^56^, ^61^, ^62^, ^63^ These pathways all converge in the downstream activation of the master regulator peroxisome‐proliferator‐activated‐receptor gamma coactivator 1‐alpha (PGC‐1α).^54^, ^56^, ^57^, ^64^ Subsequently, PGC‐1α activates nuclear respiratory factors 1–2 (NRF1‐2), which promote transcription of nuclear encoded mitochondrial proteins but also activate mitochondrial transcription factor A (tFAM) which promotes transcription of mitochondrial DNA.^54^, ^56^, ^57^, ^64^, ^65^ The majority of the evidence indicates that exercise induces mitochondrial biogenesis.^54^, ^66^, ^67^, ^68^, ^69^, ^70^ Most studies have demonstrated that mitochondrial quantity increases after voluntary exercise in mice.^71^ eNOS appears to be critically involved in this process, since mitochondrial biogenesis was significantly reduced in eNOS‐knock‐out mice after swimming.^72^ Conversely, other animal studies have suggested that exercise‐induced mitochondrial biogenesis is more prominent in skeletal muscle than in the heart.^73^, ^74^ The latter may suggest that mitochondrial biogenesis is more important for the early adaptation to exercise than the later stages.

*Mitophagy* is the opposite of mitochondrial biogenesis as it refers to the removal of damaged or dysfunctional mitochondria.^56^ The canonical mitophagy pathway is driven by the loss of mitochondrial membrane potential which fosters mitochondrial accumulation of PTEN‐induced kinase 1 (PINK1),^56^, ^57^, ^75^ and subsequent cytoplasmic‐to‐mitochondrial translocation of the E3 ubiquitin ligase Parkin. The Parkin‐mediated ubiquination of mitochondrial proteins that ensues, targets mitochondria for engulfment by autophagosome.^56^, ^57^, ^75^ The balance between mitochondrial biogenesis and mitophagy is tightly regulated through PGC‐1α by Parkin‐interacting substrate (PARIS), which prevents a mismatch in generation and degradation of mitochondria.^56^, ^57^, ^75^ Theoretically, exercise‐induced biogenesis would be accompanied by similar increases in mitophagy,^56^ to remove old and damaged mitochondria and to make room for the newly produced ones.^76^ One research group describes several studies suggesting that mitophagy was enhanced and associated with the beneficial effects of exercise.^68^ However, others reported that mitophagy was suppressed rather than stimulated.^77^, ^78^ These discrepancies are at least in part explained by the short‐lived mitophagy process in combination with the paucity of suitable models to study mitophagy *in vivo*.

*Mitochondrial dynamics* is a subtler quality control mechanism that controls the shape and size of the mitochondrial population through *mitochondrial fission and fusion* events.^79^ Dynamic interactions between mitochondria and changes in the outer mitochondrial membrane allow for continuous exchange of mitochondrial content and changes the size and shape of these organelles.^79^, ^80^, ^81^ Fission of a mitochondrion results in multiple, smaller, mitochondria, a process which is controlled by the dynamin‐1‐like protein (DRP1), mitochondrial fission 1 protein (Fis1) and mitochondrial fission factor (MFF).^80^, ^81^ Fission and the generation of fragmented mitochondria is required for mitosis, programmed cell death and mitophagy to occur.^80^, ^81^ On the other hand, fusion causes mitochondria to fuse into larger and longer mitochondria.^80^, ^81^ The functional purpose is therefore also different; fusion allows for mixing of mitochondrial content, improvement of mitochondrial function, and precedes mitochondrial biogenesis.^80^, ^81^ Critical regulators of the fusion process are mitofusin 1 and 2 (Mfn1‐2) and optical atrophy protein 1 (OPA1).^80^, ^81^ Physiological exercise appears to activate fission and fusion processes simultaneously. Coronado et al.^77^ demonstrated that increased fission is essential for the cardiac adaptation to exercise. Other authors did not detect major changes in fission in aged rats but did detect an increase in exercise‐induced fusion activity.^82^ Yoo et al.^78^ did not observe changes in mitochondrial fission and fusion after acute exercise, despite detecting enhanced cardiac mitochondrial function. Taken together, relatively little and inconsistent data are available related to the role of mitochondrial dynamics in response to exercise and require further exploration.

### Mitochondrial adaptations to exercise in skeletal muscle

The metabolic demand of skeletal muscle can increase by 100‐fold during maximal exercise,^58^ making skeletal muscle highly dependent on mitochondrial function as well^66^, ^83^, ^84^, ^85^ (*Figure* 1*C*). While the absolute mitochondrial content of skeletal muscle is low compared to the heart,^83^ it contains large mitochondrial networks – the mitochondrial reticulum – which allows for unparalleled plasticity.^66^, ^83^, ^85^, ^86^ Studies indicate that *mitochondrial biogenesis* is activated by both acute and chronic exercise.^66^, ^83^, ^85^, ^87^, ^88^ For instance, mitochondrial biogenesis is enhanced in human muscles after low‐volume high‐intensity interval training,^89^ as well as after chronic endurance exercise.^90^ The majority of evidence also suggests that acute and chronic exercise promotes Parkin‐dependent and Parkin‐independent *mitophagy* pathways.^83^, ^84^, ^85^, ^87^, ^88^, ^91^, ^92^ *Mitochondrial fission* is crucial for exercise‐induced mitochondrial adaptation,^66^, ^85^ and Fis1‐knock‐out mice develop swollen mitochondria with reduced cristae density in the gastrocnemius muscle and have reduced exercise capacity when subjected to exhaustive exercise.^66^, ^83^, ^84^, ^85^, ^86^, ^87^, ^88^, ^93^ Similarly, DRP1‐deficient mice also display deficient exercise capacity and a maladaptive response to endurance exercise.^94^ The evidence regarding fusion responses to exercise remain limited.^66^, ^84^, ^85^, ^95^

Taken together, the beneficial effects of exercise in cardiac and skeletal muscle are mediated by specific signal transduction pathways, which initiate muscle growth. In addition, mitochondrial performance is increased through orchestrated changes in mitochondrial biogenesis, mitophagy and potentially mitochondrial dynamics as well.

## Exercise in heart failure

### Pathophysiology of exercise intolerance in heart failure and the benefits of exercise

**Figure 2:**
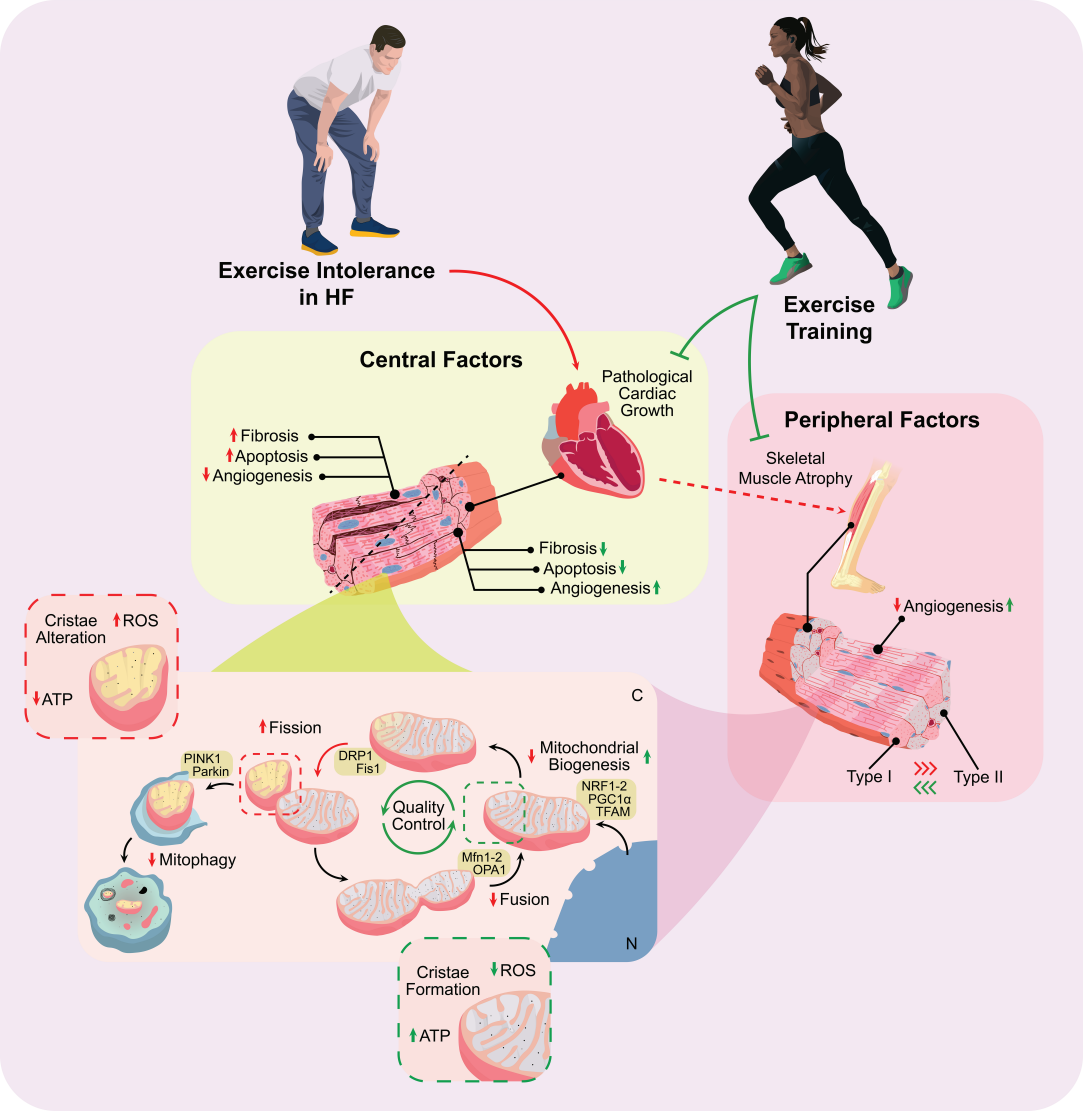
Mechanistic underpinnings of exercise intolerance in heart failure (HF) and the adaptive effects of exercise. Exercise intolerance can develop due to central and/or peripheral factors, which often include pathological cardiac remodelling and mitochondrial dysfunction. These processes can be attenuated by performing exercise, which causes adaptive effects in a disease setting in both cardiac and skeletal muscle. The mechanisms involved include growth signalling as well as mitochondrial quality control. Colours have been used structurally: red indicates effects in HF, green indicates effects of exercise training. Full lines indicate direct effects, dashed lines indicate indirect effects. ATP, adenine nucleotide triphosphate; C, cytoplasm; DRP1, dynamin‐1‐like protein; Fis1, mitochondrial fission 1 protein; Mfn1‐2, mitofusin 1 and 2; N, nucleus; NRF1‐2, nuclear respiratory factors 1 and 2; OPA1, optical atrophy protein 1; PGC‐1α, peroxisome‐proliferator‐activated‐receptor gamma coactivator 1‐alpha; PINK1, PTEN‐induced kinase 1; ROS, reactive oxygen species; tFAM, mitochondrial transcription factor A.

Exercise intolerance has a multifactorial origin,^4^, ^96^ and can be attributed to maladaptive central and peripheral factors. However, exercise itself can also exert beneficial effects in the setting of HF (*Figure* 2).

#### Central pathways underlying exercise intolerance in heart failure

*Centrally*, cardiac and pulmonary reserves are deteriorated in HF and lead to symptoms such as fatigue and dyspnoea during exercise.^4^, ^97^ HF is associated with maladaptive or pathological cardiac hypertrophy that develops in response to pathological stimuli such as hypertension or myocardial infarction.^30^, ^98^, ^99^, ^100^, ^101^, ^102^ Pathological hypertrophy, while initially compensatory, eventually fails to sustain cardiac function and often deteriorates into HF.^30^, ^98^, ^99^, ^100^, ^101^, ^102^ Molecularly, gene expression is shifted towards the foetal gene expression programme^98^, ^99^, ^100^, ^101^, ^102^ and metabolically, substrate utilization is shifted from fatty acids towards increased glucose oxidation as a compensatory response.^98^, ^99^, ^100^, ^101^, ^102^, ^103^ Contrary to physiological hypertrophy; fibrosis, apoptosis and necrosis also occur in response to pathological stimuli.^98^, ^99^, ^100^, ^101^, ^102^ Together this results in the structural and functional impairments of cardiac muscle that ultimately culminate in the HF syndrome.^29^, ^104^

In addition to structural defects, bioenergetic insufficiency is also thought to contribute to cardiac dysfunction and exercise intolerance in HF.^104^, ^105^ Cardiac mitochondrial dysfunction can be attributed to many factors, including abnormal mitochondrial ultrastructure, improper mitochondrial dynamics and functional impairments related to enhanced reactive oxygen species (ROS) and decreased ATP production.^104^ Mitochondrial fission is increased, but Parkin‐dependent mitophagy is dysregulated, resulting in increased numbers of fragmented mitochondria that are not efficiently removed through mitophagy.^104^, ^105^ Fusion and biogenesis are downregulated, further compromising mitochondrial quality.^104^, ^105^ Together, both pathological cardiac remodelling and mitochondrial dysfunction may result in unmet cardiac energy demands at rest and subsequently in response to exercise (*Figure* *2*).

#### Peripheral factors underlying exercise intolerance in heart failure

*Peripherally*, HF has been associated with several defects in the structure and function of skeletal muscle including but not limited to atrophy, an unfavourable switch to glycolytic muscle fibre types and general mitochondrial dysfunction.^4^, ^104^, ^106^, ^107^, ^108^ Furthermore, adaptive capacity of skeletal muscle is also blunted in patients with HFrEF and HFpEF.^97^ In contrast to the heart, skeletal muscle mass deteriorates in HF leading to muscular atrophy rather than hypertrophy. Bioenergetics are also impaired in skeletal muscle, which can be attributed to reductions in oxidative muscle fibres (type I) and in mitochondrial volume and density^4^, ^97^, ^104^, ^106^, ^107^, ^108^, ^109^ (*Figure* *2*).

The mechanisms causing reduced mitochondrial content and function remain relatively unexplored. Molina et al.^110^ detected reductions in mitochondrial content in skeletal muscle of older HFpEF patients, associated with diminished citrate synthase activity and lower Mfn2 levels, which were all correlated with parameters for exercise intolerance. This suggests that reduced mitochondrial fusion also contributes to skeletal muscle wasting in HF. Tsuda et al.^111^ discovered increased mitochondrial protein acetylation, associated with dysregulated fatty acid oxidation and decreased exercise capacity in a murine HF model. Hence, post‐translational modifications may play a role in the deterioration of skeletal muscle function.

#### Benefits of exercise in central and peripheral factors underlying exercise intolerance in heart failure: evidence from animal studies

Interestingly, exercise training appears to be beneficial for cardiac function in rodent models of HF.^112^, ^113^, ^114^, ^115^ For example, Campos et al.^113^ also demonstrated that 8 weeks of moderate intensity running training in rats post‐myocardial infarction (MI) HF, improved left ventricular (LV) function, associated with improvements in mitochondrial oxidative capacity. Similarly, 4 weeks of treadmill exercise improved LV function and reduced cardiac fibrosis in rats post‐MI. Mechanistically, mitochondrial biogenesis is clearly upregulated by exercise, suggesting that improvements in mitochondrial function could underlie the salutary effects.^112^ Swimming training has been shown to have similar effects, accompanied by reduced fibrosis and apoptosis, improved mitochondrial dynamics and reduced ROS production.^115^ Interestingly, endurance training prior to ischaemia/reperfusion improved mitochondrial dynamics, reduced infarct sizes and diminished pathological cardiac remodelling, suggesting that exercise has cardioprotective properties as well.^114^ Taken together, exercise training exerts beneficial effects in the HF setting, both related to pathological cardiac remodelling and mitochondrial quality control (*Figure* *2*).

Skeletal muscle responses to exercise in HF are also generally adaptive and beneficial.^116^, ^117^, ^118^, ^119^ Cai et al.^116^ demonstrated that 4 weeks of resistance and endurance training alleviated oxidative stress, protein breakdown and myocyte atrophy to the same extent. The beneficial effects of these exercise regimens were associated with activation of growth factors IGF1 and neuregulin‐1. Eight weeks of voluntary wheel running in a model of genetic heart disease, also showed that exercise reduced oxidative stress and structural damage to skeletal muscle.^117^ Bacurau et al.^118^ demonstrated the protective role of endurance exercise in HF mice, in which activation of the IGF1‐Akt–mTOR pathway prevented skeletal muscle atrophy. On the other hand, Moreira et al.^119^ demonstrated that mitochondrial citrate synthase activity was enhanced and tFAM expression was restored by endurance exercise, indicating that mitochondrial biogenesis remained intact. Hence, these studies show that skeletal muscle atrophy in HF can be overcome by exercise, possibly through beneficial effects on skeletal muscle mitochondria (*Figure* *2*; Box 1).

Box 1 **Exercise in a bottle?**

The future promise to replace physical exercise by a drug or supplement can be questioned. Can we promise that we can replace physical exercise by a tablet or bottle? Taking into account the paradigm shift toward iron supplementation and ketone administration, exercise in a bottle is what we ultimately thrive for. Whether this is a realistic gesture remains unanswered. Considering that physical exercise does much more than activating a specific pathway or growth hormone: the cyclic stretch, the increase blood flow, the vasodilation: it is hard to conceive that this is all to come out of one bottle. Tackling all the aspects of exercise is non‐realistic, unfortunately, this is something many physician‐scientists do hope for. Most probably opportunities lie more within therapeutic interventions in supplement to exercise training, rather than mimicking exercise itself.

### A novel method to quantify exercise intolerance in heart failure

In humans, the golden standard for assessing exercise capacity entails the cardiopulmonary exercise test which is widely available.^120^, ^121^ However, peripheral aspects of exercise intolerance can be studied in more detail using magnetic resonance spectroscopy (MRS) with an in‐magnet ergometer.^122^, ^123^, ^124^ With the use of 31 phosphorous (31P) MRS, *in vivo* skeletal muscle metabolism can be brought to light.^122^, ^125^ Measurements of phosphocreatine (PCr), inorganic phosphate (P_i_), ATP and phosphomonoester resonances during exercise and post‐exercise recovery allow for the determination of cellular muscle bioenergetics in the larger skeletal muscles.^122^ Additionally, oxidative muscle fibres can be visualized, and mitochondrial content can be estimated.^126^ Changes in metabolite measurements such as the P_i_/PCr ratio during incremental exercise correlate well with the degree of exercise intolerance in HF patients.^127^, ^128^ Furthermore, measurement of PCr resynthesis during recovery is considered to be a reliable estimate of mitochondrial functioning in skeletal muscle, because PCr resynthesis is predominantly dependent on oxidative mitochondrial ATP synthesis.^124^ For instance, this technique was applied by van der Ent et al.^128^ who have investigated forearm skeletal muscle metabolism in chronic HF patients and provided evidence for decreased mitochondrial oxidative capacity, resulting in a decreased exercise adaptive capacity in HF patients. Weiss et al.^96^ provided the evidence that both HFrEF and HFpEF patients exhibited normal basal skeletal muscle metabolism, but in response to exercise, skeletal muscle metabolism was disrupted. This was characterized by rapid decrements in phosphates and low oxidative capacity (Box 2).

Box 2 **A novel golden standard to determine exercise intolerance?**

Many clinical trials utilize the 6‐minute walk test and/or Kansas City Cardiomyopathy Questionnaire to determine exercise capacity. In clinical practice, cardiopulmonary exercise testing is however golden standard. For research purposes this often is too laborious and costly but would provide more insight into specific parameters of i.e., peak oxygen uptake, which are more accurate and of prognostic value for cardiovascular outcome. Recent studies using a 31 phosphorous surface coil in Magnetic Resonance Spectroscopy with an in‐magnet ergometer, have shown to provide detailed functional status of peripheral skeletal muscles. This technique offers more specific insight into mechanistic deficits in heart failure patients with exercise intolerance. Yet its feasibility and cost‐effectiveness should be considered. Perhaps, the development of a patient‐specific panel or flow chart of tests could provide an individualized status of exercise tolerance, which could serve as a more accurate golden standard in both a research and clinical setting.

## Possibilities to improve exercise intolerance in heart failure

### Benefits of endurance exercise in heart failure: evidence from human studies

**Figure 3:**
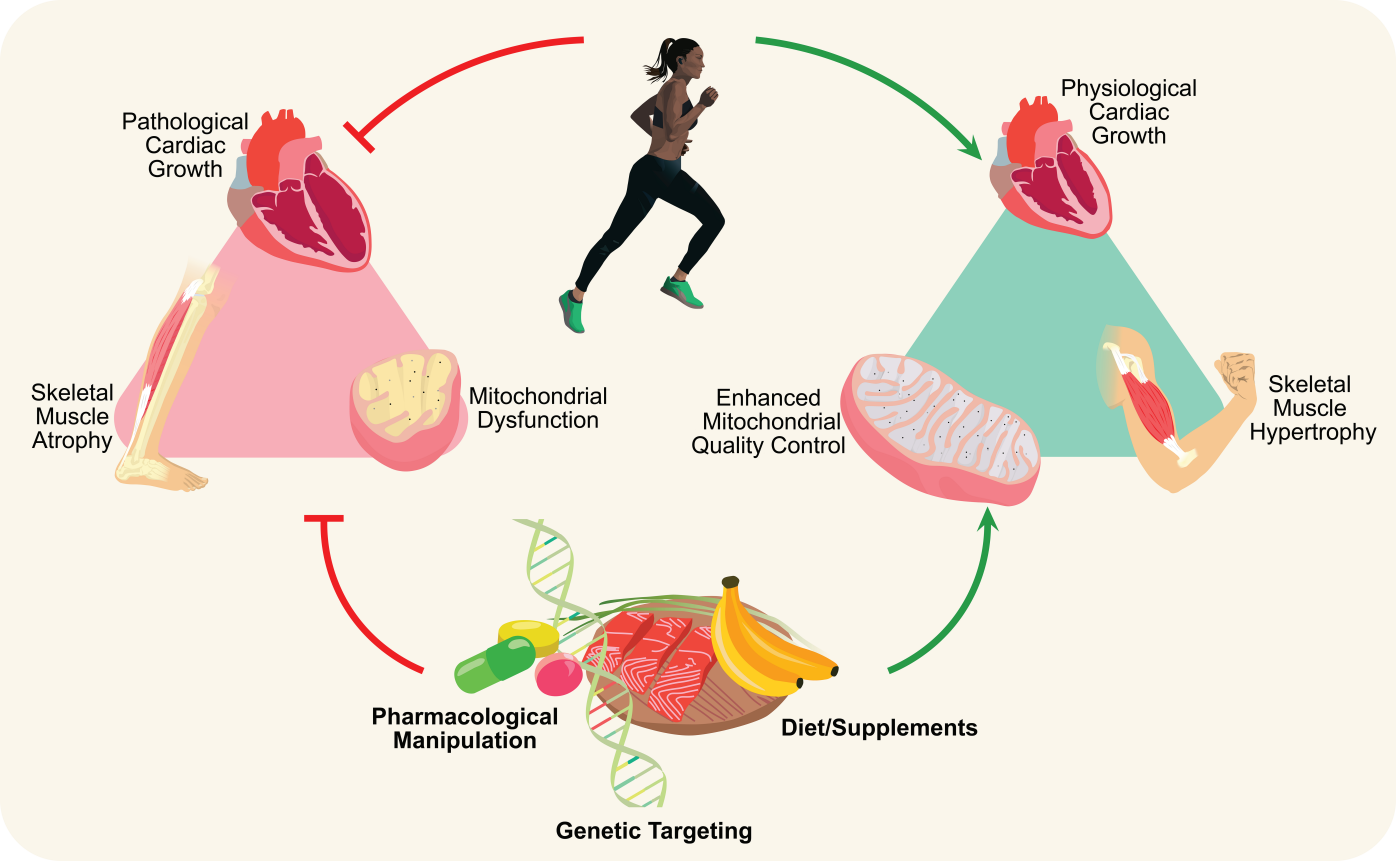
Exercise as a molecular therapy for heart failure‐associated exercise intolerance. The adaptive effects of exercise in both health and disease may provide therapeutic targets to improve exercise intolerance in heart failure. The underlying mechanistic pathways involve stimulation of physiological cardiac growth, skeletal muscle hypertrophy and enhanced mitochondrial quality control. This is accompanied by inhibition of pathological cardiac growth, skeletal muscle atrophy and mitochondrial dysfunction. These exercise‐induced effects can be enhanced by targeting these pathways with pharmacological manipulation, diets/supplements and/or genetic targeting.

Clinical data also suggest that exercise is beneficial for HF patients. In fact, multiple studies including randomized controlled clinical trials such as the HF‐ACTION trial, systematic reviews and meta‐analyses have shown that exercise is safe and improves symptoms in HF patients, both HFrEF and HFpEF patients.^5^, ^6^, ^129^, ^130^, ^131^, ^132^, ^133^, ^134^, ^135^, ^136^, ^137^, ^138^, ^139^, ^140^, ^141^, ^142^, ^143^, ^144^ Exercise training is therefore recommended in HF patients.^5^, ^16^ Unfortunately, exercise is initially accompanied by increases in discomfort and many HF patients are apprehensive or physically challenged by non‐cardiac comorbidities. Long‐term adherence to such regimens is therefore low.^16^, ^18^, ^145^ Accordingly, nutriceutical or pharmacological strategies to boost exercise performance in HF patients could be key. Based on the evidence presented in this review, we believe that there are multiple targets and strategies that should be explored to improve exercise performance in HF (summarized in *Figure* *3*).

### Targeting growth in cardiac and skeletal muscle

There are clear distinctions in the molecular growth responses between physiological and pathological growth in cardiac and skeletal muscle. One might therefore argue that it would be beneficial to activate physiological pathways in HF patients, i.e. to superimpose physiological growth responses in a pathological setting. A potential focus should be the activation of exercise‐induced signalling pathways, such as targeting factors in the Akt‐C/EBPß‐CITED4 pathway. Considering that this pathway signals through miRs, RNA‐based therapeutics may offer opportunities to promote physiological growth in HF.^19^, ^23^, ^44^, ^146^, ^147^ Another example, of a potential target to regulate physiological growth is A‐kinase interacting protein 1 (AKIP1).^148^, ^149^, ^150^ Studies with neonatal rat ventricular cardiomyocytes demonstrated an induction of cardiomyocyte hypertrophy by overexpression of AKIP1.^149^ This hypertrophic response was associated with activation of Akt signalling without enhanced expression of pathological gene markers,^149^ suggesting a role for AKIP1 in physiological cardiomyocyte hypertrophy. Activation of such signalling pathways could lead to beneficial reprogramming and could improve exercise performance. In skeletal muscle, it is also of importance to stimulate physiological growth, but most specifically, novel targets should be developed to overcome skeletal muscle atrophy. The knowledge regarding resistance and endurance training is increasing, and this provides improvements in skeletal muscle mass, potentially through mechanisms involving Akt and mTORC1.^118^

### Boosting mitochondrial performance in cardiac and skeletal muscle

Current literature describes several cardioprotective roles of exercise due to underlying beneficial mitochondrial mechanisms, suggesting potential targets to improve exercise tolerance in HF.^151^, ^152^, ^153^ Stimulation of these mitochondrial adaptive responses including mitochondrial biogenesis, mitophagy and mitochondrial dynamics could potentially also improve exercise capacity in HF patients. It is however important to consider that cardiac dysfunction may not be restored solely by improving mitochondrial performance.^154^ In skeletal muscle, improvements in mitochondrial quality control could enhance the adaptive response to stress and block or reverse the unfavourable muscle fibre switching. Recently, our department uncovered an important role of the erythropoietin receptor in skeletal muscle, a novel target which is critical for mitochondrial biogenesis in skeletal muscle and the response to physiological exercise.^155^ Potentially, stimulating skeletal muscle erythropoietin signalling could benefit for patients with mitochondrial myopathies, skeletal muscle fatigue, or atrophy.

## Conclusion

Exercise intolerance is the central and most debilitating symptom in HF patients and limited therapies are available. Evidence is emerging that exercise training has beneficial effects on both cardiac and skeletal muscle, in health and disease settings. Therefore, in order to overcome exercise intolerance in HF patients, the primary advice remains exercise. However, this should be accompanied by additional therapies to alleviate initial symptoms associated with exercise in HF patients. We do propose that novel molecular therapies to boost muscle growth and mitochondrial quality control in HF should always be combined with some form of exercise training. Possibilities for such supplemental molecular therapies lie within pharmacological, genetic and/or dietary targeting of pathways associated with adaptive effects of exercise in cardiac and skeletal muscle. Hence, increased understanding of exercise‐associated growth signalling, and mitochondrial quality control mechanisms may broaden the horizon for exercise as a therapy for HF. Future studies should focus on unravelling the mechanistic underpinnings of exercise‐induced benefits in both cardiac and skeletal muscle.
